# PUPMCR: an R package for image-based identification of color based on Rayner’s (1970) terminology and known fungal pigments

**DOI:** 10.1093/biomethods/bpaf004

**Published:** 2025-01-12

**Authors:** Niña Rose Zapanta, Rhenz Hannah Santos, Jericho Ivan Pineda, Jireh Sealtiel Pedrosa, Kristine Joyce Rabelas, Charina Samontan, Lourdes Alvarez, Chester Deocaris

**Affiliations:** Department of Biology, College of Science, Polytechnic University of the Philippines, Sta. Mesa, Manila, 1016, Philippines; Department of Biology, College of Science, Polytechnic University of the Philippines, Sta. Mesa, Manila, 1016, Philippines; Department of Biology, College of Science, Polytechnic University of the Philippines, Sta. Mesa, Manila, 1016, Philippines; Department of Physical Science, College of Science, Polytechnic University of the Philippines, Sta. Mesa, Manila, 1016, Philippines; Department of Physical Science, College of Science, Polytechnic University of the Philippines, Sta. Mesa, Manila, 1016, Philippines; Department of Physical Science, College of Science, Polytechnic University of the Philippines, Sta. Mesa, Manila, 1016, Philippines; Department of Biology, College of Science, Polytechnic University of the Philippines, Sta. Mesa, Manila, 1016, Philippines; Department of Physical Science, College of Science, Polytechnic University of the Philippines, Sta. Mesa, Manila, 1016, Philippines

**Keywords:** fungi, morphology, color, CIELAB, RGB, Euclidean, Chi-square

## Abstract

Fungi are eukaryotic organisms grouped based on different traits of their morphology. In 1970, R. W. Rayner published *A Mycological Colour Chart* to provide a standardized system for identifying color in fungi. While its terminologies have contributed a standard way of color matching for taxonomic diagnoses, this method using the personal color perception of the observer does not guarantee accuracy. Considering the diversity of fungi, visual color matching is expected to be challenging without a standard assisting instrument. In this study, the R package PUPMCR is developed to approximate the color name and associated pigments of fungal species based on the pixel coordinates of its uploaded image. This software utilizes CIELAB and RGB color spaces as well as Euclidean and Chi-square distance metric systems. The package is tested and validated using 300 fungal images as a dataset for conducting interrater reliability tests. Results showed the highest agreement for parameters utilizing the RGB color space (Cohen’s kappa values: 0.655 ± 0.013 for RGB and Euclidean; 0.658 ± 0.004 for RGB and Chi-square), attributed to its computational efficiency, which facilitates more uniform binning and universally scaled distance metrics. The produced color-identifying tool is also available as a Shiny web application (https://pupmcr.shinyapps.io/PUPMCR/) to allow better accessibility for users on the World Wide Web. The development of PUPMCR not only benefits a variety of users from its free accessibility but also provides a more reliable color identification system in the field of mycology.

## Introduction

Fungi are a diverse group of organisms estimated to have 2–11 million species across different biomes [1]. However, the total number of fungal species accurately described and characterized is only about 150 000 [2]. Such diversity of this clade is evident in the plentitude of morphological characteristics investigated by scientists, including their colors. Relatively, colors in fungi are also associated with their produced secondary metabolites, such as melanin, carotenoid, azaphilones, and polyketides, giving species distinct characteristics and uses [3].

In 1970, R. W. Rayner created *A Mycological Colour Chart* to provide a standardized system for the color identification of fungi. It contains over a thousand color names based on Dade’s chart with corresponding Munsell notations. This chart is one of many color charts created to provide a systematic color-naming system for fungal species. However, while Rayner terminologies provided a more standard way of visual color matching for taxonomic diagnosis, this method using the personal color perception of the observer does not guarantee accuracy.

Notably, the use of traditional color charts generally poses disadvantages, including inaccessibility and inevitable quality degradation. Sources like Rayner’s (1970) are excessively rare and expensive, and mycologists tend to utilize materials only available to them. Given the nature of printed books, color samples are also prone to changes, such as fading, depending on the dyes used for printing.

Considering the diversity of fungi, the colors of such species are expected to vary significantly, making visual color matching challenging, especially without a standard assisting instrument. Importantly, color matching has been constrained by categorization methods as existing tools often rely on subjective classification or expensive equipment [4].

R is a statistical programming software popular among biologists due to its user-friendly and open-source features. There are R packages created for image analysis and color quantification [5, 6], but there is currently none that caters to fungal photographs. Additionally, color profiling methods of images are not usually available in multiple color spaces [4] or distance methods.

With the problems presented by the color chart methods, the PUPMCR (Polytechnic University of the Philippines Mycological Chart Rayner) R package was created. It describes and matches colors of fungal species with over 1252 Rayner terminologies by binning pixels found in an uploaded image. Aside from color terms, PUPMCR also provides descriptions of hue groups and associated pigments of fungi, while also generating a plot coordinate and a histogram for each image analysis.

The package offers two options for color spaces: (1) CIELAB (luminance, red-green, and blue-yellow), which is perceptually uniform and not dependent on devices, and (2) RGB (red, green, and blue), which is mainly optimized for digital displays. Two distance metrics are also available for color matching, namely Euclidean and Chi-square.

This study aims to provide a standardized toolbox for color identification in mycology by facilitating the description of fungi based on their colors and potential associated pigments. Furthermore, the development of the PUPMCR Shiny app is intended to offer a user-friendly color identification tool for those who may not be familiar with the R programming environment.

## Materials and methods

### Lookup table

**Figure 1.**
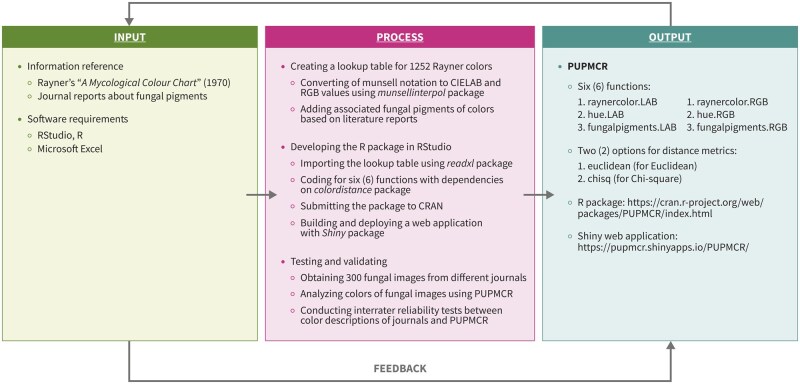
Input–process–output flowchart for the development of PUPMCR

PUPMCR references the book *A Mycological Color Chart* by R. W. Rayner, published in 1970. Specifically, a total of 1252 Munsell notations from the chart were converted to CIELAB and RGB values to be used as data for the package. Moreover, various journal articles and reviews were used as informational sources for fungal pigments. The development of PUPMCR was performed in RStudio, R version 4.2.3, and utilized several R packages described in the flowchart process (Fig. 1).

### Lookup table

**Figure 2.**
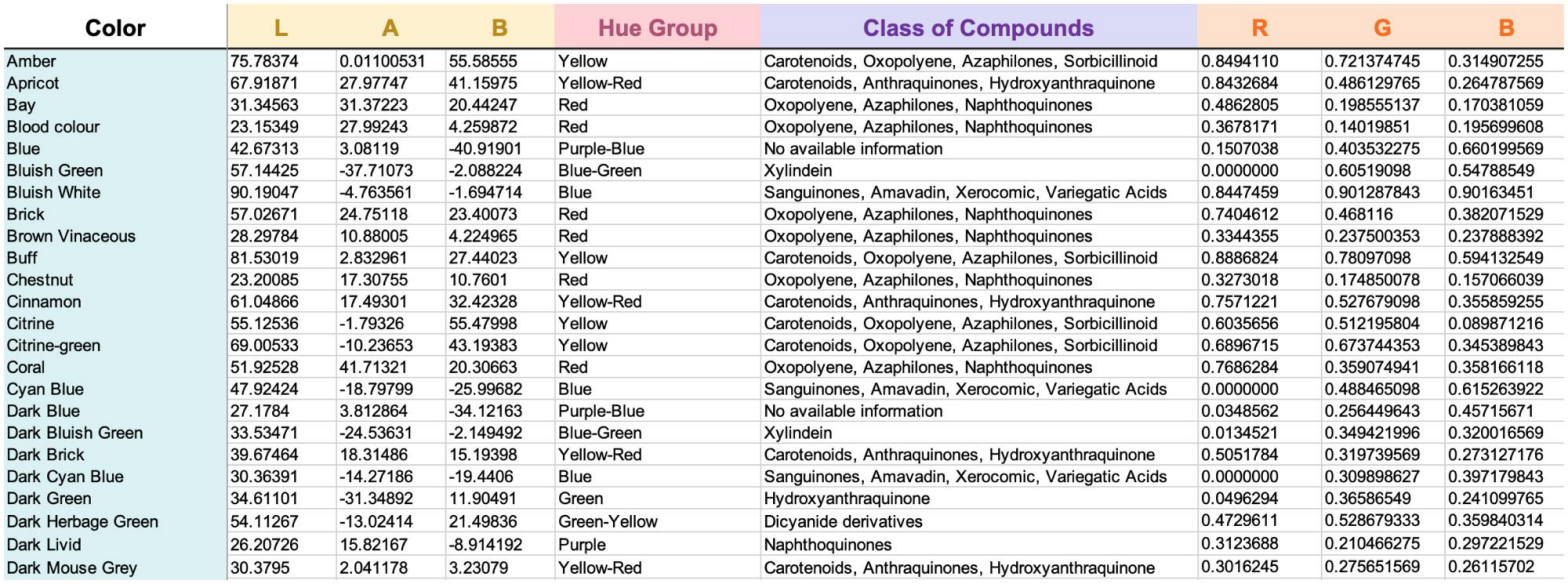
Preview of the constructed reference lookup table for PUPMCR containing 1252 Rayner color nomenclatures (Color), CIELAB color coordinates (L, A, B), hue group (Hue Group), pigments association (Class of Compounds), and RGB color coordinates (R, G, B). This file is available on the tar.gz file of the R package

A lookup table (Fig. 2) was constructed with the first column containing the names of 1252 Rayner colors; the second, third, and fourth columns providing the L*, a*, and b* color coordinates, accordingly; the fifth and sixth column describing the hue group and pigments of the Rayner colors; and R, G, B color coordinates on the seventh, eighth, and ninth columns, accordingly.

Additionally, the *munsellinterpol* [7] package, which comes with munselltoLab and munselltoRGB functions, was utilized to convert the Munsell equivalents of Rayner colors in the color chart to LAB and RGB values. The lookup table was then imported to RStudio and integrated into the package using the *readxl* [8] package.

Meanwhile, related pigments assigned to different Rayner colors were collected from review articles about fungal pigments [9–11] and were assigned manually to every Rayner Colors based on their hue group.

### Package details

PUPMCR has six exported functions listed in Table 1. The development of the package relied on several R packages but with the main dependency on *colordistance* [5] for image analysis and data clustering. Mainly, colordistance functions such as loadimage, plotPixels, getLabHist, and getImageHist were used and integrated into the color matching methods of PUPMCR.

**Table 1.** PUPMCR functions and descriptions

Function	Description
raynercolor.LAB	Generates results for color names from the CIELAB color space
raynercolor.RGB	Generates results for color names from the CIELAB color space
hue.LAB	Generates results for hue groups based on the CIELAB color matches
hue.RGB	Generates results for hue groups based on the RGB color matches
fungalpigments.LAB	Generates results for associated fungal pigments or class of compounds based on the CIELAB color matches
fungalpigments.RGB	Generates results for associated fungal pigments or class of compounds based on the RGB color matches

Each function generates a unique result based on the color space used and the specific data extracted from an image of a fungi. It has two parameters: (i) the image being analyzed and (ii) the color distance used, which can be either euclidean (for Euclidean) or chisq (for Chi-square).

In PUPMCR, the Euclidean distance method is the default color matching metric. Hence, an image analysis for detecting color names with CIELAB and Euclidean parameters can be done by running the following line of code in the R console:

> raynercolor.LAB(“fungi.png”)

Meanwhile, for Chi-square, the distance method must be specified:

> raynercolor.LAB(“fungi.png”, distance.method = chisq)

The main workflow of PUPMCR consists of the following steps:

- *Image preparation*. Colored fungal images in JPEG or PNG are obtained, and image backgrounds are masked using an editor outside R.
- *Color matching*. Images of fungi are imported in R as 3D arrays, and non-background pixels are grouped into color categories, resulting in a normalized color space histogram. Pixel color coordinates are then compared from the reference lookup table using the defined distance method.
- *Results generation*. The top eight results (color names, hues, or pigments) of the analysis are shown alongside their return color values, 3D pixel plot, and color histogram.

**Figure 3.**
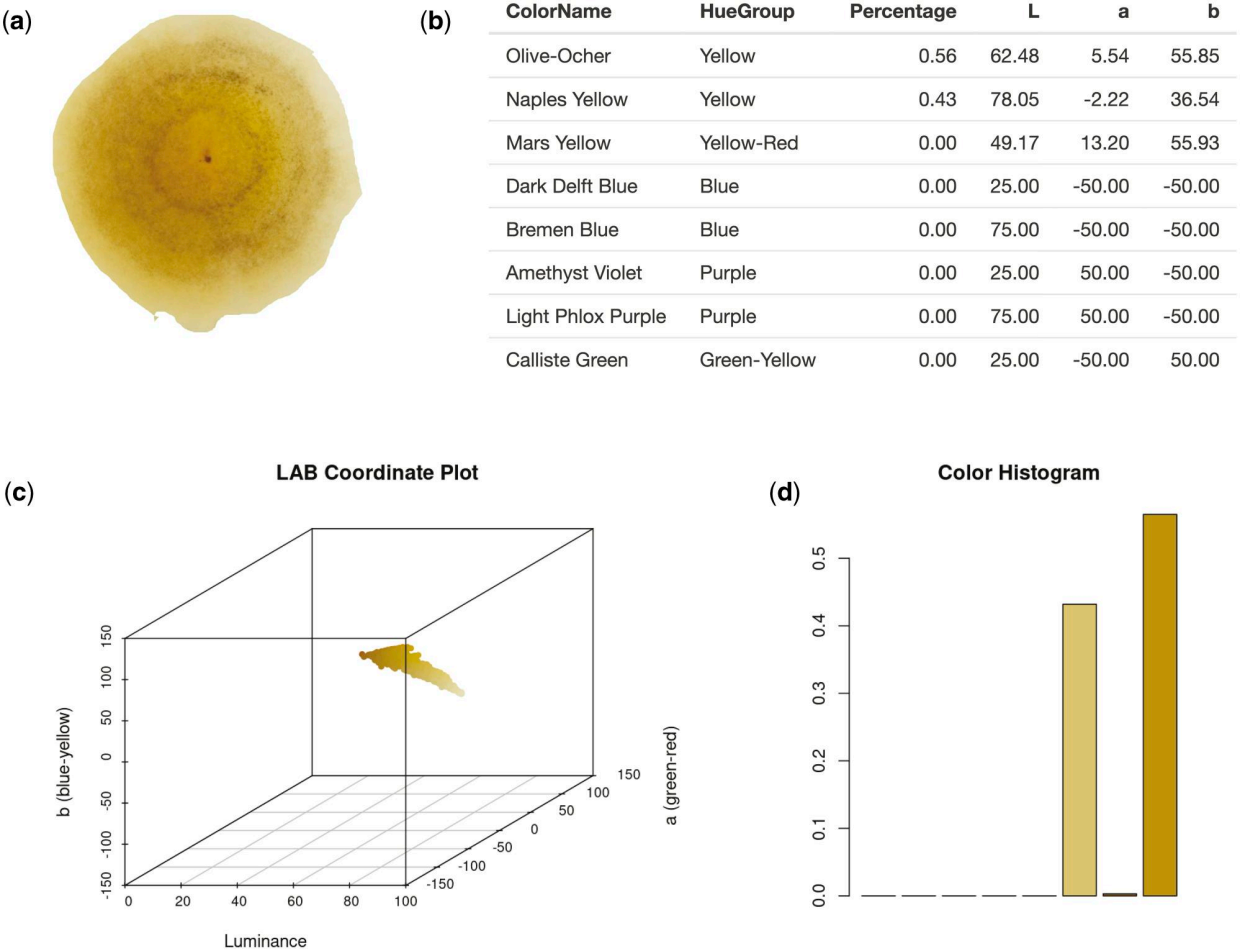
Analysis results of PUPMCR for an uploaded (**a**) fungal image: (**b**) top eight color names, (**c**) 3D plot of all non-background pixels in CIELAB color space, and (**d**) color histogram showing pixel proportions for eight bins

Figure 3 shows the results generation of PUPMCR. Before importing the fungal image, its background is first masked out or removed. The default upper and lower bounds (RGB [0, 1, 0]) from the *colordistance* package were used for PUPMCR, which works well for images with a bright green background. Additionally, the reference white light color was set to D65 CIE standardized illuminant, ideal for images taken with an average daylight source.

The raynercolor.LAB function displays the names of colors in a photograph based on nomenclatures defined by Rayner and uses the CIELAB color space for matching. When using this function, it is required to provide (i) the path to the background-masked image and (ii) the distance method for the analysis (Euclidean is the default and not necessary to type if chosen).

> library(PUPMCR)

> raynercolor.LAB(“fungi.png”)

The same parameters are needed to implement the rest of all PUPMCR functions, including hue. LAB and fungalpigments. LAB, which generates the hue groups and associated pigments of top detected colors in an image using CIELAB, respectively.

> hue.LAB(“fungi.png”)

> fungalpigments.LAB(“fungi.png”, distance.method = chisq)

Each fungal image imported results in a data frame ranging from qualitative (color names, hues, or pigments) to quantitative (the average value of pixels in a bin as well as the percentage or proportion of pixels assigned to that bin).

> raynercolor.LAB(“fungi.png”)

**Table:** 

	L	a	b	Pct	ColorName
1	25.00000	−50.000000	−50.00000	0.000	Dark Delft Blue
2	75.00000	−50.000000	−50.00000	0.000	Bremen Blue
3	25.00000	50.000000	−50.00000	0.000	Amethyst Violet
4	75.00000	50.000000	−50.00000	0.000	Light Phlox Purple
5	25.00000	−50.000000	50.00000	0.000	Calliste Green
6	78.27409	−2.237716	36.44494	0.441	Naples Yellow
7	49.46682	12.774178	56.28875	0.003	Mars Yellow
8	62.93410	5.253878	55.14371	0.556	Olive-Ocher

As such, the olive-ocher color of fungi falling into bin 8 has a 62.93 luminance (L; on a 0–100 scale) and relatively not skewing on the red-green (a) channel (5.25 on a −100 to 100 scale) but on the blue-yellow (b) channel, located more toward the yellow end (55.14 on a −100 to 100 scale). The pixels make up 55.6% of the fungal photograph.

The package development of PUPMCR further utilized other R packages, such as *roxygen2* [12] and *devtools* [13], to build all documentation required and simplify tasks like package installation, documentation creation, testing, and version control integration, respectively. The PUPMCR package can be downloaded for free at https://cran.r-project.org/package=PUPMCR.

### Development of the PUPMCR shiny application

**Figure 4.**
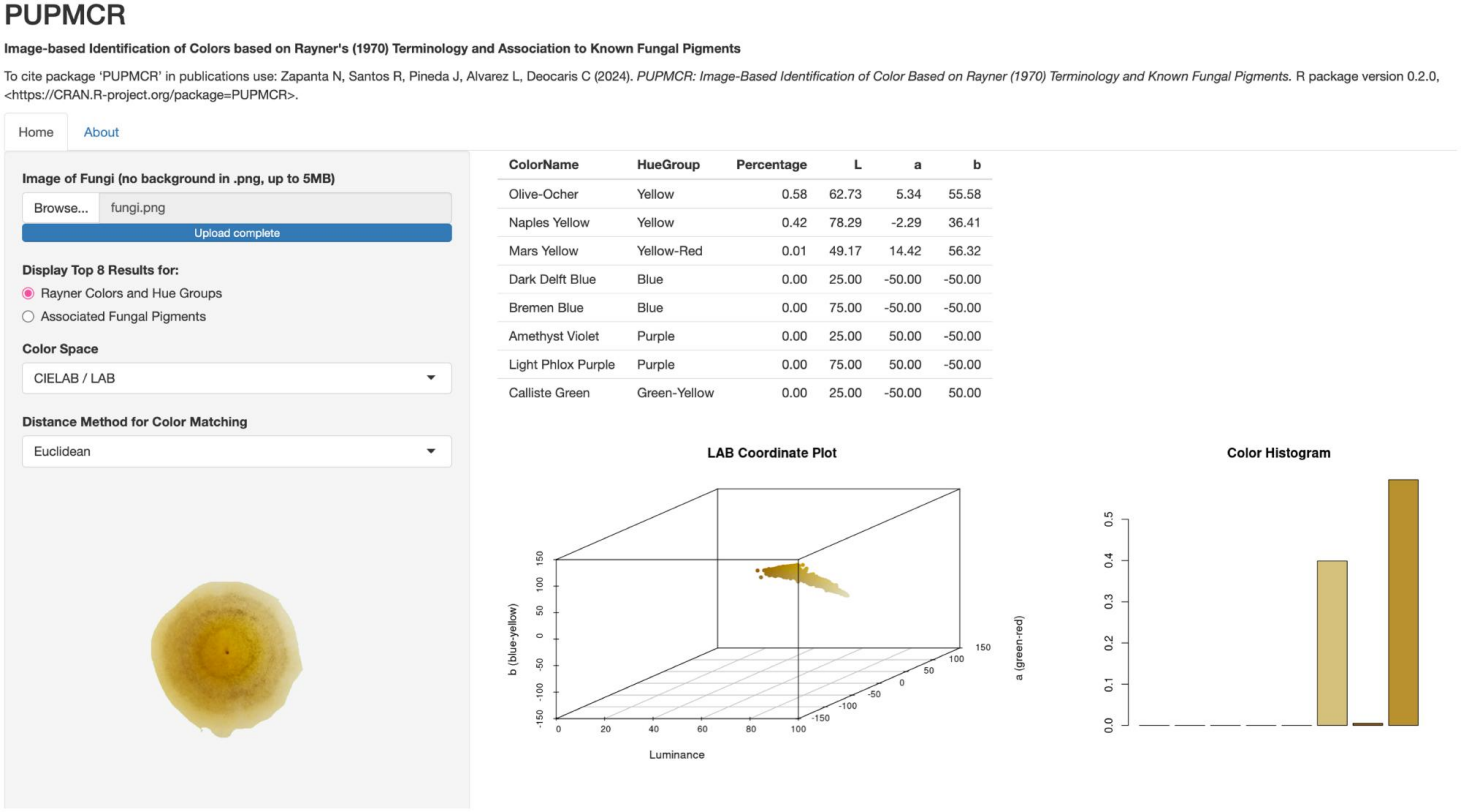
Overview of the PUPMCR Shiny app deployed using shinyapps.io

The PUPMCR R package was used to create a corresponding Shiny web application (Fig. 4). This app was developed using the *shiny* [14] package and deployed through shinyapps.io. It presents the functionalities of the package, with a better user interface and accessibility outside the R program.

The app allows image analysis without the need to type codes in an R console, but instead, just click parameter options in the side panel. As such, users can still choose between the two metric systems for color matching, Euclidean and Chi-square, and between the two available color spaces, CIELAB and RGB, in determining the top results for Rayner color names, hue groups, or associated pigments of a fungal image.

Similar to the R package, a pixel coordinate plot and a histogram from the color analysis are displayed. However, an additional display for the uploaded image of fungi is included in the Shiny app to enhance visualization during analysis. All these features provide a straightforward and user-friendly app for anyone interested in fungal color identification. The PUPMCR Shiny app can be accessed through the link https://pupmcr.shinyapps.io/PUPMCR/.

### Testing and validation

Using the *irr* [15] package, confusion matrices for the combination of each color space and distance method of PUPMCR were generated. Each matrix has a sample size of 300 fungal images obtained from various mycology journal articles listed in Table 2. The sample size of 300 was chosen to capture a broad range of fungi species, colors, and pigment variations. Relatively, the criteria for selecting photographs from journals include the use of the Rayner color charts in describing colors and that pictures are presented in color instead of black and white. This sets the comparison between color descriptions perceived by human eyes, specifically by the authors, and PUPMCR.

**Table 2.** Journal sources for generating the confusion matrix

Title	Author/s	Genera/species of fungi	No. of images obtained
Back to the roots: a reappraisal of *Neocosmospora*	Sandoval-Denis *et al*. Lombard [16]	*Neocosmospora* spp.	60
*Corynespora*, *Exosporium* and *Helminthosporium* revisited—New species and generic reclassification	Voglmayr and Jaklitsch [17]	*Helminthosporium* spp.	14
*Didymellaceae* revisited	Chen *et al*. [18]	*Allophoma* spp., *Ascochyta* spp., *Boeremia* spp., *Didymella* spp., *Epicoccum* spp., *Heterophoma* sp., *Neoascochyta* sp., and *Neodidymelliopsis* sp.	33
Diversity and taxonomy of *Chaetomium* and chaetomium-like fungi from indoor environments	Wang *et al*. [19]	*Amesia* spp., *Arcopilus* spp., and *Botryotrichum* spp.	10
First report of *Phyllosticta citricarpa* and description of two new species, *P. paracapitalensis* and *P. paracitricarpa*, from citrus in Europe	Guarnaccia *et al*. [20]	*Phyllosticta* spp.	12
Genera of phytopathogenic fungi: GOPHY 4	Chen *et al*. [21]	*Allophoma* sp., *Alternaria* spp., *Elsinoe* spp., and *Seiridium* spp.	18
Highlights of the *Didymellaceae*: A polyphasic approach to characterise *Phoma* and related pleosporalean genera	Aveskamp *et al*. [22]	*Phoma* spp., and *Boeremia* spp.	6
New and Interesting Fungi 6	Crous *et al*. [23]	*Polyscytalum* sp.	1
Phylogenetic lineages in the Capnodiales	Crous *et al*. [24]	*Catenulostroma* sp., *Devriesia* spp., and *Hortaea* sp.	5
Phylogeny and taxonomy of the scab and spot anthracnose fungus *Elsinoë* (*Myriangiales*, *Dothideomycetes*)	Fan *et al*. [25]	*Elsinoe* spp.	12
Phytopathogenic Dothideomycetes	Crous *et al*. [26]	*Paraconiothyrium* sp., *Pleurophoma* sp., *Aposphaeria* spp., and *Caryophylloseptoria* spp.	6
Redisposition of acremonium-like fungi in *Hypocreales*	Hou *et al*. [27]	*Acremonium* spp., *Emericellopsis* spp., *Nothoacremonium* spp., *Lasionectria* spp., *Ramosiphorum* spp., etc.	67
Revising *Clonostachys* and allied genera in *Bionectriaceae*	Zhao *et al*. [28]	*Nectriopsis* sp., *Mycocitrus* sp., and *Clonostachys* spp.	11
Revising the *Schizoparmaceae*: *Coniella* and its synonyms *Pilidiella* and *Schizoparme*	Alvarez *et al*. [29]	*Coniella* spp.	15
Taxonomy and Pathology of *Togninia* (*Diaporthales*) and its *Phaeoacremonium* Anamorphs	Mostert *et al*. [30]	*Phaeoacremonium* spp.	15
Wood staining fungi revealed taxonomic novelties in *Pezizomycotina*: New order *Superstratomycetales* and new species *Cyanodermella oleoligni*	van Nieuwenhuijzen *et al*. [31]	*Superstratomyces* spp. and *Cyanodermella* spp.	12
Mycosphaerella is polyphyletic	Crous *et al*. [32]	*Readeriella* sp.	1
Delimiting *Cladosporium* from morphologically similar genera	Crous *et al*. [33]	*Verrucocladosporium* sp.	1
The Genera of Fungi—G6: Arthrographis, Kramasamuha, Melnikomyces, Thysanorea, and Verruconis	Hernández-Restrepo *et al*. [34]	*Verruconis* sp.	1

The confusion matrix is a multiclass type containing hue group categories: yellow, green, red, blue, and purple (Supplementary data). Expected values came from the published color descriptions of the dataset, deduced according to their hue based on Munsell notations, while predicted values came from the results generated by PUPMCR. Color analysis for the 300 images was done five times to establish reliability. The matrices were used to evaluate the performance of the package in terms of accuracy: [(TP + TN)/(TP + TN + FP + FN)].

The same dataset collected from various journal articles was also subjected to the interrater reliability test using the *irr* package (Supplementary data). Four interrater tests were conducted, which correspond to the pairing for each color space and distance metric. Relatively, the reliability between the two raters (paper descriptions and PUPMCR results) in each test was determined through their Cohen’s kappa values. Specifically, rater 1 refers to the color descriptions provided by journal articles for species based on manual identification using Rayner’s Mycological Chart (1970). In contrast, rater 2 refers to the color name description generated by PUPMCR.

Furthermore, the validation of pigments association by the package was done by analyzing images of representative fungal species with reported production of specific compounds. For instance, running images of yellow-red *Monascus* sp. associated with azaphilones, and blue-green *Chlorociboria* sp. linked to xylindein, among others. It is noteworthy that this was only possible for species with a quality image available on the internet and literature sources and that some hue groups have no known pigments associated with them at the moment, limiting the validation method.

## Results and discussion

### Interrater reliability testing

Four confusion matrices were generated to quantify the accuracy of PUPMCR in terms of hue group detection and its level of agreement with literature reports. The tests generally demonstrated a moderate agreement for all parameters except for the combination of CIELAB and Chi-square (Table 3). Notably, hue mismatches are specifically low for yellow (0.326 ± 0.006) and purple (0.366 ± 0.031) in the same set of parameters.

**Table 3.** Accuracy and Cohen’s kappa value of PUPMCR across different parameters.

	CIELAB		RGB	
	Euclidean	Chi-square	Euclidean	Chi-square
Yellow	0.889 ± 0.008	0.326 ± 0.006	0.901 ± 0.005	0.899 ± 0.003
Green	0.922 ± 0.005	0.851 ± 0.021	0.945 ± 0.005	0.942 ± 0.005
Red	0.928 ± 0.006	0.861 ± 0.006	0.924 ± 0.003	0.927 ± 0.005
Blue	0.951 ± 0.018	0.835 ± 0.021	0.943 ± 0.028	0.977 ± 0.014
Purple	0.963 ± 0.017	0.366 ± 0.031	0.959 ± 0.027	0.927 ± 0.015
Cohen's kappa value	0.605 ± 0.013	0.035 ± 0.007	0.655 ± 0.013	0.658 ± 0.004
Interpretation	Moderate	None	Moderate	Moderate

Several factors may contribute to the limited compatibility of the CIELAB color space with the Chi-square distance measure. First, CIELAB is designed to be a perceptually uniform color space where the Euclidean distances between points represent perceived color differences more accurately than RGB space [35]. Meanwhile, the Chi-square distance is a statistical measure that assesses the difference between two probability distributions [36]. As such, it emphasizes the comparative distributions of color intensity rather than absolute perceptual differences, which does not suit the continuous representation of the CIELAB color space and leads to discrepancies when applied to such data.

Furthermore, the nature of color representation in CIELAB involves an interaction of lightness, chroma, and hue, which may not be adequately captured when employing Chi-square distances. For instance, yellow presented a significantly low mismatch (0.326 ± 0.006), suggesting that while CIELAB can generally represent yellow well, the Chi-square method may not account for the subtleties of color variation in this space. This limitation could be attributed to the inherent sparsity of yellow across the color spectrum, which might not align with the assumptions of distribution used in the Chi-square calculation.

Also, potential issues may arise from how color data is binned or quantified before employing the Chi-square method. The discretization of color values into bins can introduce noise and misrepresent the actual continuous color data. Although this distance method usually works effectively, it can lead to larger color distances when images feature similar colors that are categorized differently, since it considers the bins to be independent of one another [4]. As such, images that are all-black and all-gray may have the same distance as images that are all-black and all-white.

This misrepresentation can disproportionately affect the performance of the Chi-square method when evaluating the similarities or differences in color, further exacerbating the weak agreement observed between these two parameters. For this reason, the Euclidean metric is set as the default distance method for PUPMCR.

Moreover, when interpreting the Cohen's kappa values, a low kappa (0.035 ± 0.007) indicates an almost negligible agreement when using CIELAB with Chi-square distances. This suggests that the categorical interpretation of color in the context of Chi-square distances fails to translate effectively from the metric-based analysis of CIELAB, which is more nuanced.

To obtain the best possible result for color identification, the CIELAB color space must be utilized for datasets with inconsistent lighting or average daylight lighting (D65) since the L in CIELAB measures the lightness value of a given image, separating it from color. Meanwhile, the RGB color space must be employed for datasets with consistent lighting, given that it is an additive model that links lightness value with color intensities. Euclidean is generally the more suited metric distance for both color spaces due to its stable and direct method of measuring, compared to Chi-square distance, which is best for color histogram comparison.

Future work may benefit from exploring alternative distance metrics or enhancing methods of data representation in PUPMCR to improve hue detection accuracy across various color spaces and statistical measures.

### Pigments agreement

**Figure 5.**
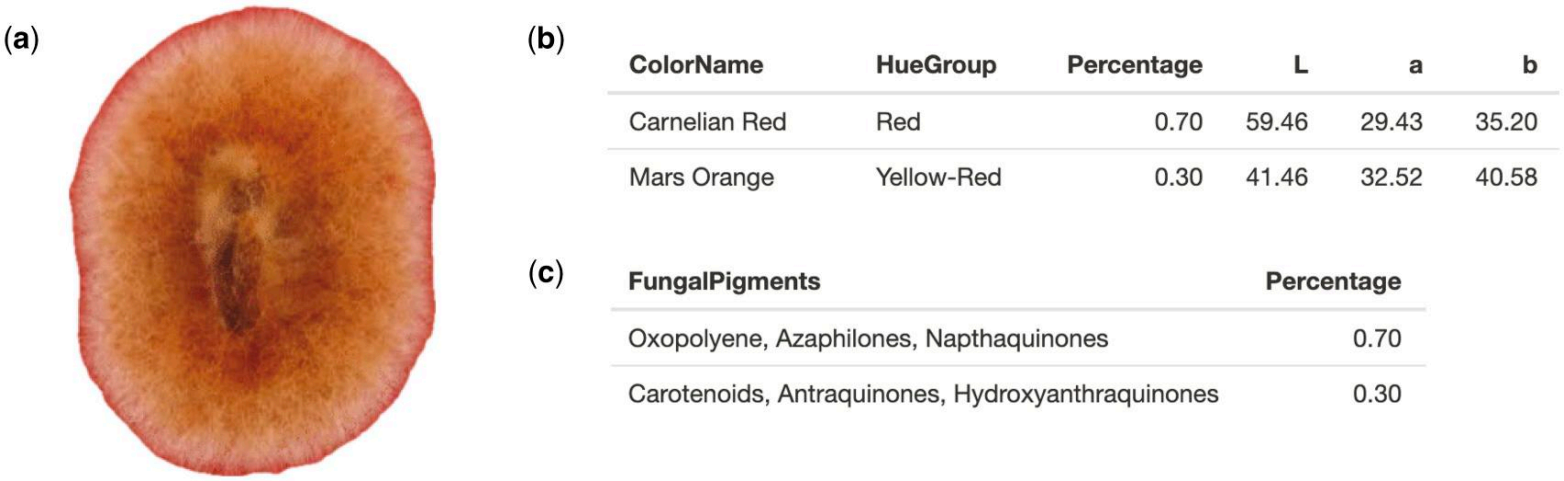
*Monascus purpureus* (**a**) sample on RBA plate and its generated PUPMCR results for CIELAB and Euclidean parameter, (**b**) color names and hue groups, and (**c**) fungal pigments. Fungal photograph reproduced from [38] under a CC BY-NC license.

To measure the ability of PUPMCR in detecting hue and associated pigments, images of select fungal species with reported production of pigments described in the lookup table were used. For instance, *Monascus purpureus* is a filamentous fungus belonging to the genus Monascus known to produce a complex mixture of three pigment categories: orange, red, and yellow, each with two components of polyketide origin. These are secondary metabolites with a common azaphilone skeleton [37]. PUPMCR results for a sample of *M. purpureus* on rose Bengal agar (RBA) plate [38] showed a general agreement with its reported red to yellow-red hue and pigment association with azaphilone (Fig. 5).

**Figure 6.**
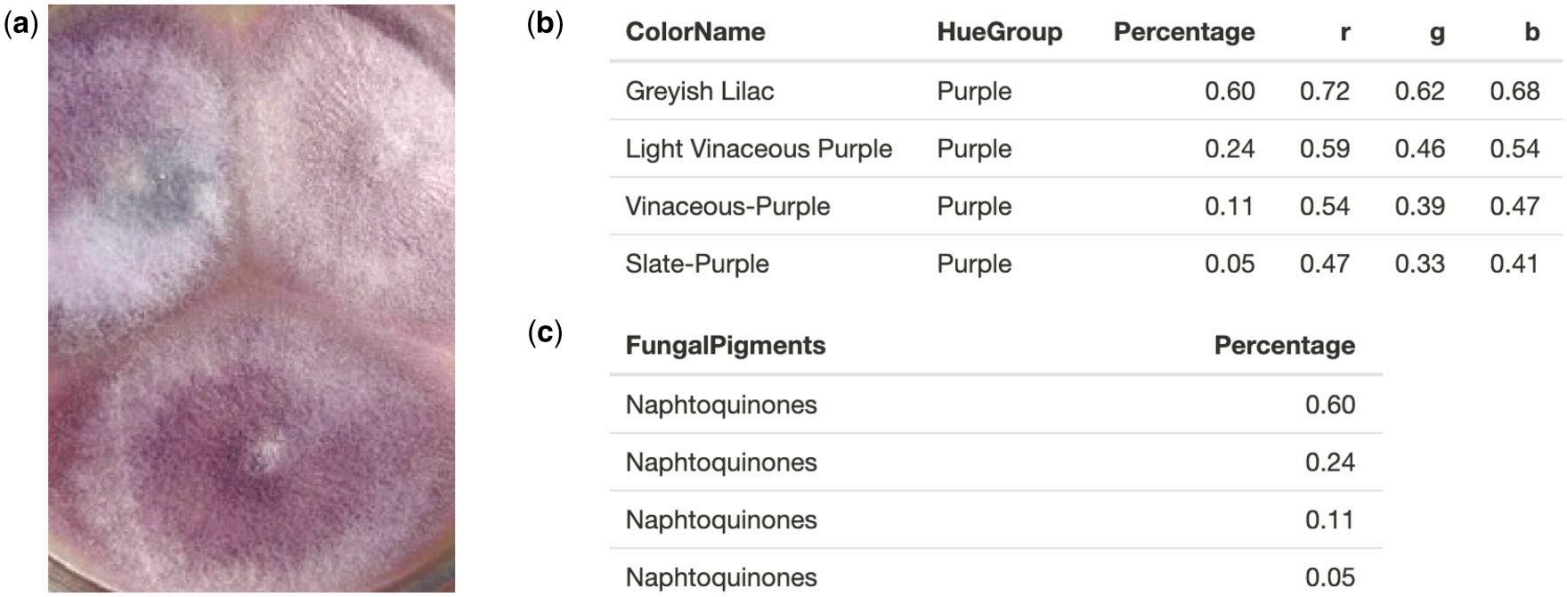
*Fusarium proliferatum* (**a**) sample on PDA and its generated PUPMCR results for RGB and Euclidean parameter, (**b**) color names and hue groups, and (**c**) fungal pigments. Fungal photograph reproduced from [39] under a CC BY-NC license.

Similarly, *Fusarium proliferatum*, reported to produce several compounds, including beauvericin, enniatins, fusaric acid, and naphthoquinone pigments [39], was analyzed. PUPMCR results of its colony on potato dextrose agar (PDA) demonstrated a general agreement with its reported purple hue and fungal pigment association with naphthoquinone (Fig. 6).

**Figure 7.**
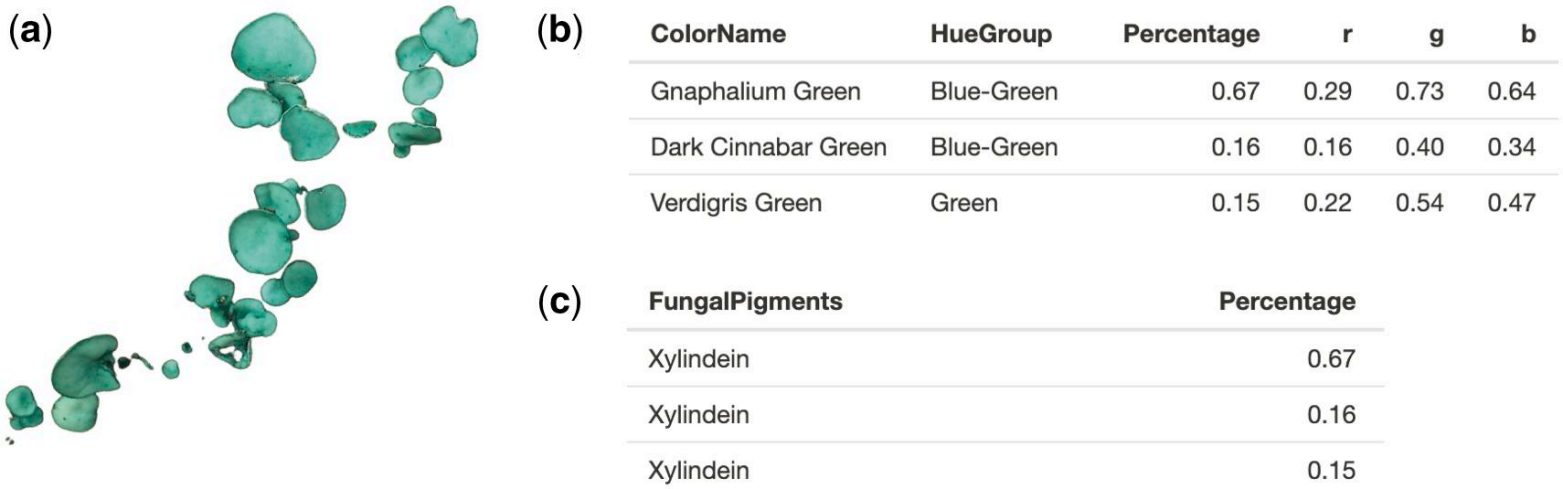
*Chlorociboria aeruginascens* (**a**) fruiting body and its generated PUPMCR results for RGB and Chi-square parameter, (**b**) color names and hue groups, and (**c**) fungal pigments. Fungal photograph reproduced from [40] under a CC BY 3.0 license.

Moreover, the fruiting body of *Chlorociboria aeruginascens* [40], a species known to produce a blue-green dimeric naphthoquinone derivative pigment called xylindein, was run in the package. PUPMCR results indicated that it has a blue-green hue and pigment association with xylindein, fitting the descriptions found in the literature (Fig. 7).

**Figure 8.**
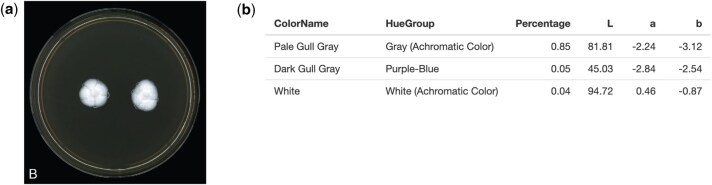
*Nothoacremonium vesiculophorum* (**a**) sample and (**b**) its generated PUPMCR results for CIELAB and Euclidean parameter. Fungal photograph reproduced from [27] under a CC BY-NC-ND license.

Fungal species of achromatic colors were also tested in the package, which generally revealed the significance of lighting in its color detection. In the case of *Nothoacremonium vesiculophorum*, reported to be entirely white [27], a high percentage resulted in gray due to dominating shadows in the image (Fig. 8). For this reason, it is advised that uploaded photos must have little to no shadows to produce more accurate results.

**Figure 9.**
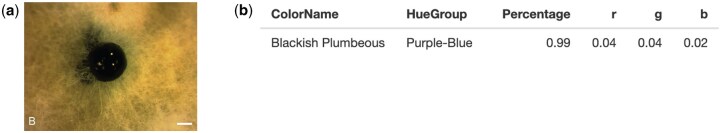
*Melanconis italica* (**a**) sample on RBA and (**b**) its generated PUPMCR results for RGB and Euclidean Parameter. Fungal photograph reproduced from [41] under a CC BY-NC-ND license.

Furthermore, a conidioma of *Melanconis italica*, described as black [41], was used to test the ability of the package in detecting such color. PUPMCR generated blackish plumbeous as its top color and purple-blue as its hue. While some colors of the media surroundings were reflected from the glistening surface of the fungal structure, this did not affect the overall reading of the package for black (Fig. 9).

Notably, running black images in the PUPMCR generally result in blackish plumbeous or any similar colors rather than true black with the achromatic hue. This is because most devices utilize liquid crystal display technology with a backlight that inhibits pixels from entirely cutting off transmitted light from global dimming. This often leads to screens not achieving an actual dark/black state [42].

### PUPMCR shiny application

**Figure 10.**
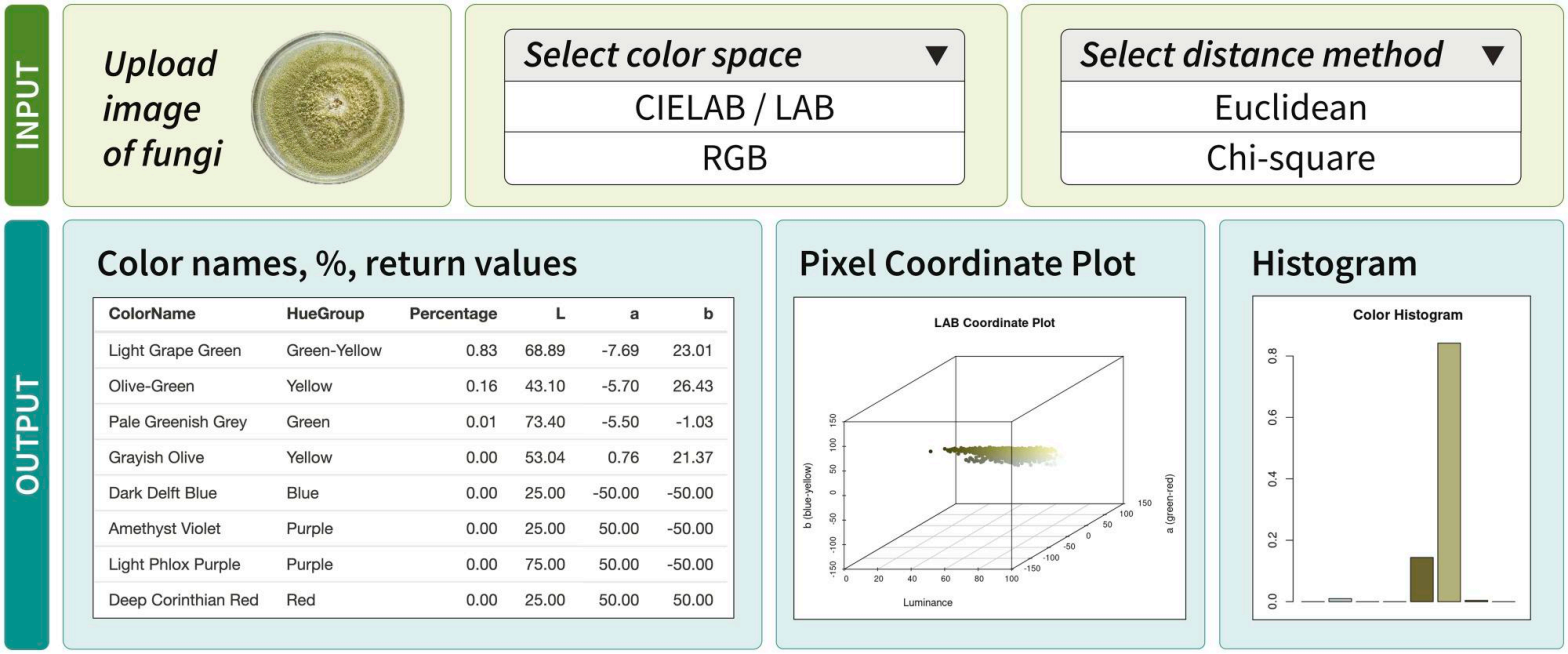
Overview of features of the PUPMCR Shiny application. Inputs include uploaded fungal image, and parameters, such as color space and distance method. Outputs comprise a table of the top eight color names, percentages, and return color values of detected pixels in an image, a pixel coordinate plot, and a color histogram.

The PUPMCR Shiny app provides an accessible and user-friendly interface for analyzing fungal images and their color properties, mirroring the functionalities of the PUPMCR R package. Key enhancements in the Shiny app include interactive features that cater to both novice and experienced users and facilitate efficient data visualization (Fig. 10).

Users can upload a fungal image in .*png* format (no background, less than 5 MB), making it easy to analyze color distributions. The app allows users to select parameters for analysis, specifically the color space (CIELAB or RGB) and the distance method (Euclidean or Chi-square). These choices significantly affect the analysis results and can be tailored depending on the preferences and research objectives of users.

Upon processing the uploaded image, the app displays the top eight colors along with their corresponding hue groups or fungal pigments. This output table includes the percentage and return values of the color notations, allowing users to understand the dominant colors in the image. As such, the fungal image in Fig. 10, with a dominant green-yellow color, exhibited a high percentage of light grape green (83%) pixels.

In addition, the Shiny app generates visualizations: (1) a pixel plot coordinate graph illustrating the position of colors in the uploaded image within the chosen color space, offering insights into color relationships; and (2) a color histogram depicting the distribution of the top 8 colors to provide an immediate understanding of the color composition of a fungal image. These graphs can be downloaded and saved as images by right-clicking and choosing “Save Image As…”

Importantly, users should ensure that uploaded images of fungi are in the preferred file size and format (<5 MB; .*png* or .*jpeg*) to avoid errors in the website. Same errors are shown in R when using the package and corrupted/incorrect files are uploaded.

## Discussion

PUPMCR addresses the need for a standardized and user-friendly color identification tool in the field of mycology. The package provides a method for describing colors and potentially associated pigments in an objective and repeatable way without requiring the use of expensive and inaccessible equipment. The results generated by PUPMCR ensure enhanced and time-efficient descriptions based on a standard mycological color chart.

Notably, choosing the appropriate color space and distance method for analysis affects the produced results of the package. The Euclidean distance is set as the default parameter for the distance method due to its straightforward approach in matching color pixels from images to the ones found in the reference lookup table. As such, results of the interrater reliability testing showed moderate agreement for tests involving Euclidean paired with both CIELAB and RGB color spaces. This is opposed to Chi-square, which was inefficient when paired with CIELAB.

While the CIELAB color space is considered the superior choice for biologically relevant quantitative color analyses due to its perceptual uniformity, it is not necessarily the best option for the color identification methods of PUPMCR. The main goal of the package is to describe colors and pigments objectively, not depending on the color perception of organisms. For this reason, a color space like RGB is recommended as it is more computationally tractable, allowing for more consistent binning and universally scaled distance measurements [4].

Moreover, most digital photographs are available in the RGB format, and choosing this color space would require less time for conversions from RGB to CIELAB during image analysis. Regardless, the CIELAB option is still useful for datasets with inconsistent lighting, as its L component stands for lighting. For fungal images taken with varying or inconsistent light sources, it is recommended that users choose the CIELAB function to generate results that take lighting into account for color matching.

PUPMCR stands out as an innovative and user-friendly tool for describing fungal colors. It aims to complement traditional color-matching techniques that use standardized charts rather than serve as a replacement. Its smooth integration within the R environment allows users to utilize additional R packages and tools for an in-depth analysis of fungal images. In the future, the researchers are dedicated to improving the package by adding new pigments, refining key parameters, and developing more features for image analysis, keeping it at the cutting edge of fungal research.

## Supplementary Material

bpaf004_Supplementary_Data

## Data Availability

Project name: PUPMCR; Project home page: https://cran.r-project.org/web/packages/PUPMCR/index.html; https://pupmcr.shinyapps.io/PUPMCR/. Operating systems: Windows, Linux, Mac; Programming language: R; License: GPL-2; Any restrictions to use by non-academics: No restrictions.
